# Dehydrocostus Lactone Suppresses LPS-induced Acute Lung Injury and Macrophage Activation through NF-κB Signaling Pathway Mediated by p38 MAPK and Akt

**DOI:** 10.3390/molecules24081510

**Published:** 2019-04-17

**Authors:** Yunjuan Nie, Zhongxuan Wang, Gaoshang Chai, Yue Xiong, Boyu Li, Hui Zhang, Ruiting Xin, Xiaohang Qian, Zihan Tang, Jiajun Wu, Peng Zhao

**Affiliations:** Department of Basic Medicine, Wuxi School of Medicine, Jiangnan University, Wuxi 214122, Jiangsu, China; nieyunjuan@jiangnan.edu.cn (Y.N.); m18861807206@163.com (Z.W.); chaigaoshang@jiangnan.edu.cn (G.C.); xiongyyue@163.com (Y.X.); lby199804@163.com (B.L.); zhanghui99111@yeah.net (H.Z.); 1280116118@vip.jiangnan.edu.cn (R.X.); qianxh@rjlab.cn (X.Q.); 15061885262@163.com (Z.T.); orange.jj@foxmail.com (J.W.)

**Keywords:** dehydrocostus lactone, LPS, macrophage, acute lung injury, p38MAPK, Akt, NF-κB

## Abstract

Acute lung injury (ALI) is a severe clinical disease marked by dysregulated inflammation response and has a high rate of morbidity and mortality. Macrophages, which play diverse roles in the inflammatory response, are becoming therapeutic targets in ALI. In this study we investigated the effects of dehydrocostus lactone (DHL), a natural sesquiterpene, on macrophage activation and LPS-induced ALI. The macrophage cell line RAW264.7 and primary lung macrophages were incubated with DHL (0, 3, 5, 10 and 30 μmol/L) for 0.5 h and then challenged with LPS (100 ng/mL) for up to 8 hours. C57BL/6 mice were intratracheally injected with LPS (5 mg/kg) to induce acute lung injury (ALI) and then treated with a range of DHL doses intraperitoneally (5 to 20 mg/kg). The results showed that DHL inhibited LPS-induced production of proinflammatory mediators such as iNOS, NO, and cytokines including TNF-α, IL-6, IL-1β, and IL-12 p35 by suppressing the activity of NF-κB via p38 MAPK/MK2 and Akt signaling pathway in macrophages. The *in vivo* results revealed that DHL significantly attenuated LPS-induced pathological injury and reduced cytokines expression in the lung. NF-κB, p38 MAPK/MK2 and Akt signaling molecules were also involved in the anti-inflammatory effect. Collectively, our findings suggested that DHL is a promising agent for alleviating LPS-induced ALI.

## 1. Introduction

Acute lung injury (ALI) is a devastating respiratory disorder characterized by rapid alveolar injury, severe hypoxemia, uncontrolled inflammatory response, and cytokine accumulation, which may develop into acute respiratory distress syndrome (ARDS) [1]. Currently, there are no effective treatments for ALI/ARDS and despite substantial large-scale clinical trials and studies, it remains major causes of morbidity and mortality in clinics [2]. This means the discovery and development of effective curative candidates remains a major focus for research.

ALI development into ARDS may be started by uncontrolled inflammatory responses which are initiated, amplified and regulated by various proinflammatory molecules and cytokines produced by diversified inflammatory cells [3,4]. Recent studies have demonstrated that continuous activation of macrophages was one of the most important steps responsible for accelerating this development [5,6]. Macrophages exert distinctly exclusive functions during ALI/ARDS process through the production of proinflammatory mediators including nitric oxide (NO), chemokines and cytokines such as tumor necrosis factor alpha (TNF-α), interleukin (IL)-12, IL-6 and IL-1β [7]. Disturbing macrophage activation therefore may be a potential way to treat ALI/ARDS [8,9]. 

Lipopolysaccharide (LPS), a main component of bacterial cell walls, has been identified as a key factor in ALI/ ARDS development. Exposure to LPS causes inflammatory cell infiltration, leading to generation of cytokines and chemokines that subsequently lead to initiation and propagation of the inflammation response seen in ALI/ARDS [10]. LPS binds Toll-like receptor 4 (TLR4) on the cell surface which then stimulates the recruitment and activation of the adaptor protein myeloid differentiation primary response gene 88 (MYD88) to the receptor complex, in turn leading to the activation of intracellular pathways [11,12]. p38 MAPK has been demonstrated to be an important kinase activated by TLR4/MyD88, phosphorylation of its downstream kinases, MK2, enhances mRNA levels and stability of cytokines such as TNF-α, IL-6 and IL-12 [13,14]. Further studies have identified the p38 MAPK pathway as playing a vital role in the response to LPS-stimulated inflammation and macrophage activation [15]. Akt is another important molecule activated by the receptor complex of TLR4/MyD88 and plays a crucial role in the development of ALI/ARDS mediated by proinflammatory cytokines [16,17]. The above two signaling pathways, p38 MAPK/MK2 and Akt, can both target the nuclear transcription factor NF-κB to regulate the expression of multiple genes which is responsible for the progression of a number of inflammatory diseases [18,19]. Exploring drug candidates affecting these signaling pathways in macrophages may be a critical step towards helping to treat, and potentially cure, LPS-induced diseases.

Dehydrocostus lactone (DHL) is a natural sesquiterpene lactone derived from various medicinal plants such as *Inula helenium L.* and *Saussurea lappa* [20]. Numerous studies have found that DHL has anti-tumor properties, as well as hepatoprotective and immunomodulatory effects, and that some of these effects are associated with the downregulated activity of NF-κB mediated by P38/MK2 and Akt signaling pathway [21,22,23]. However, whether DHL has a beneficial effect on macrophage-mediated ALI has yet to be fully investigated. The present study was conducted to investigate the anti-inflammatory effect and mechanisms of DHL and explore the potential compound for treating ALI/ARDS.

## 2. Results

### 2.1. DHL Suppressed the Activation of p38/MK2 and Akt Signaling Pathway

**Figure 1:**
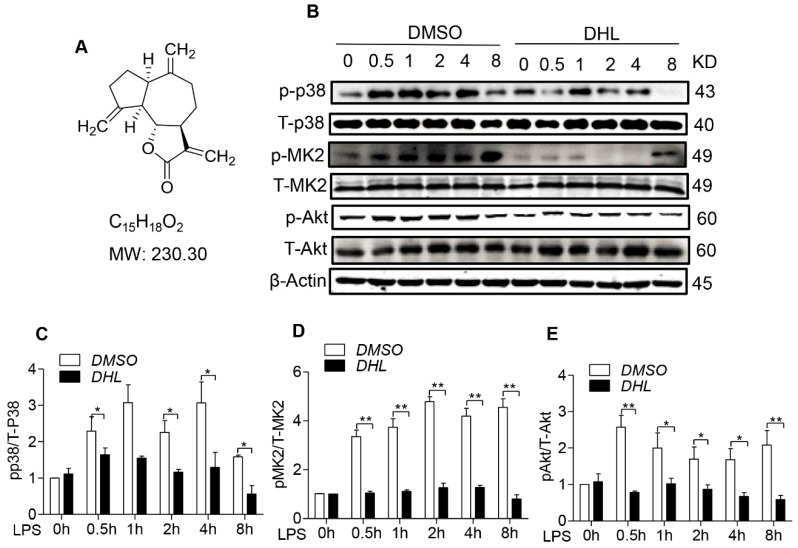
DHL reduced p38 MAPK, MK2 and Akt phosphorylation in LPS-stimulated RAW264.7 cells. (**A**) Chemical structure of DHL. RAW264.7 cells were pretreated with either 5 μmol/L DHL or 0.1% DMSO for 0.5 h before stimulation with LPS (100 ng/mL) for 0, 0.5, 1, 2, 4 and 8 h. (**B**) The protein expression levels of p-p38 MAPK, p38 MAPK, p-MK2, MK2, p-Akt and Akt were evaluated by western blotting. (**C**–**E**) Quantitative analysis of the ratios of p-p38/T-p38, p-MK2/MK2 and p-Akt/Akt normalized with β-actin was performed using Image J software. Data represents means ± SEM based on three independent experiments, * *p* < 0.05 and ** *p* < 0.01.

The chemical structure of DHL is shown in Figure 1A. Studies have shown that the activation of p38 and Akt signaling molecules play a crucial role in macrophage function and the inflammatory process [24]. Here, we investigated the role of DHL in the phosphorylation of p38 MAPK, MK2 and Akt by western blot analysis.

RAW264.7 murine macrophage cells were pretreated with 5 μmol/L DHL or 0.1% DMSO for 30 min, then challenged with LPS (100 ng/mL) for 0–8 h. LPS challenging markedly increased the phosphorylation of p-p38, p-MK2 and p-Akt, while DHL pre-incubation significantly inhibited these phosphorylation (Figure 1B–E), suggesting that DHL could inhibit the activity of p38/MK2 and Akt signaling pathways.

### 2.2. DHL Reduced the Expression of iNOS and Inhibited NO Production in both Raw264.7 Cells and Lung Macrophages

**Figure 2:**
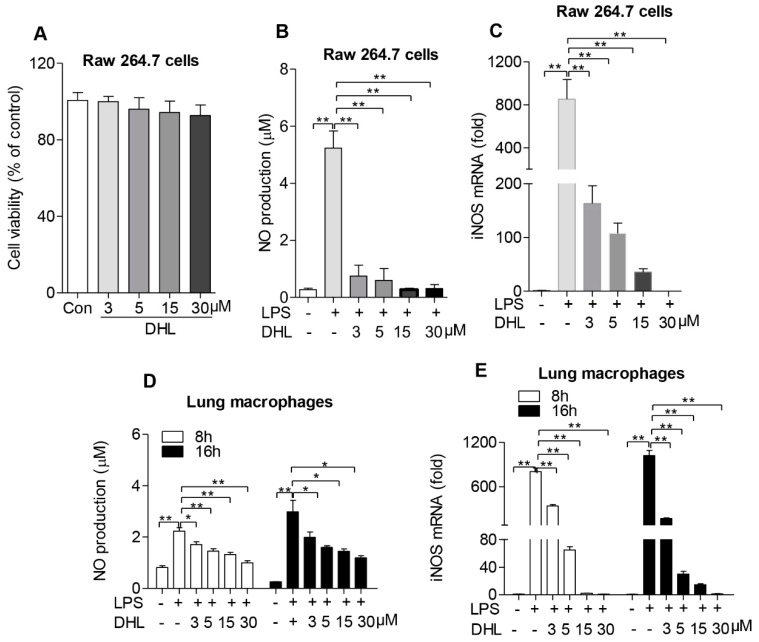
DHL inhibited NO production and iNOS expression in LPS-stimulated macrophages. (**A**) RAW264.7 cells were treated with DHL at 0, 3, 5, 10 and 30 μmol/L for 48 h, and cell viability was determined by MTT assay. (**B**,**C**) RAW264.7 cells were pretreated with 0, 3, 5, 10 and 30 μmol/L DHL for 30 mins followed by stimulation with LPS (100 ng/mL) for 24 h, the supernatant of culture medium was collected for NO detection, and the mRNA levels of iNOS were analyzed by quantitative real-time PCR. Lung macrophages were pretreated with 0, 3, 5, 10 and 30 μmol/L DHL for 30 min followed by stimulation with LPS (100 ng/mL) for 8 and 16 h, (**D**) The culture supernatants were collected and NO levels detected with a Griess reagent kit; (**E**) Cells were collected to isolate total RNA, and the mRNA levels of iNOS were analyzed by quantitative real-time PCR. The data represent mean ± SEM of three independent experiments, * *p* < 0.05 and ** *p* < 0.01.

The impact of DHL on the viability of macrophages was measured by MTT assay. The results showed that DHL had no significant adverse effect on the viability of RAW264.7 cells (Figure 2A). High levels of NO and iNOS contribute to pathophysiological inflammation [25]. Therefore, we examined whether DHL could affect the levels of NO and iNOS expression in macrophages stimulated with LPS. RAW264.7 murine macrophage cells were treated with DHL and then with LPS to stimulate inflammation. As shown in Figure 2B, with concentrations ranging from 3 to 30 μM DHL significantly inhibited the LPS-induced NO production in a dose-dependent manner. Next, we determined the level of iNOS expression, which is responsible for NO production in cells. DHL pretreatment inhibited LPS-induced iNOS mRNA level in RAW264.7 cells in a dose-dependent manner (Figure 2C). 

To further determine the effect of DHL on pathophysiological inflammation, the NO production and iNOS expression secreted by lung macrophages in response to LPS and DHL treatment were also measured. The production of NO and iNOS in lung macrophages was also shown to decrease after DHL administration (Figure 2D,E). These results suggest that DHL could suppress inflammation via inhibition of iNOS expression, thus reducing NO production.

### 2.3. DHL Inhibited LPS-Induced Proinflammatory Cytokines in both RAW264.7 Cells and Lung Macrophages

We next examined whether DHL could affect the production of inflammatory cytokines produced by activated macrophages using quantitative RT-PCR assay. 

**Figure 3:**
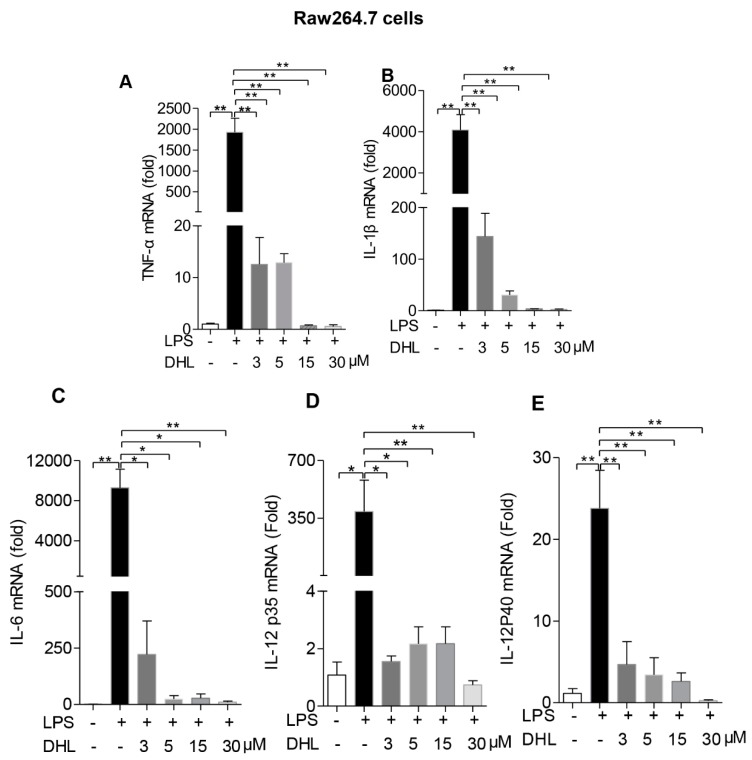
DHL decreased proinflammatory cytokine production in LPS-stimulated RAW264.7 cells. RAW264.7 cells were pre-treated with 0, 3, 5, 10 and 30 μmol/L DHL for 0.5 h before LPS (100 ng/mL) for 24 h, cells were collected to isolate total RNA, the mRNA expression level of (**A**)TNF-α, (**B**) IL-1β, (**C**) IL-6, (**D**) IL-12 p35 and (**E**) IL-12 p40 were measured by quantitative PCR. Values represent means ± SEM, * *p* < 0.05 and ** *p* < 0.01.

**Figure 4:**
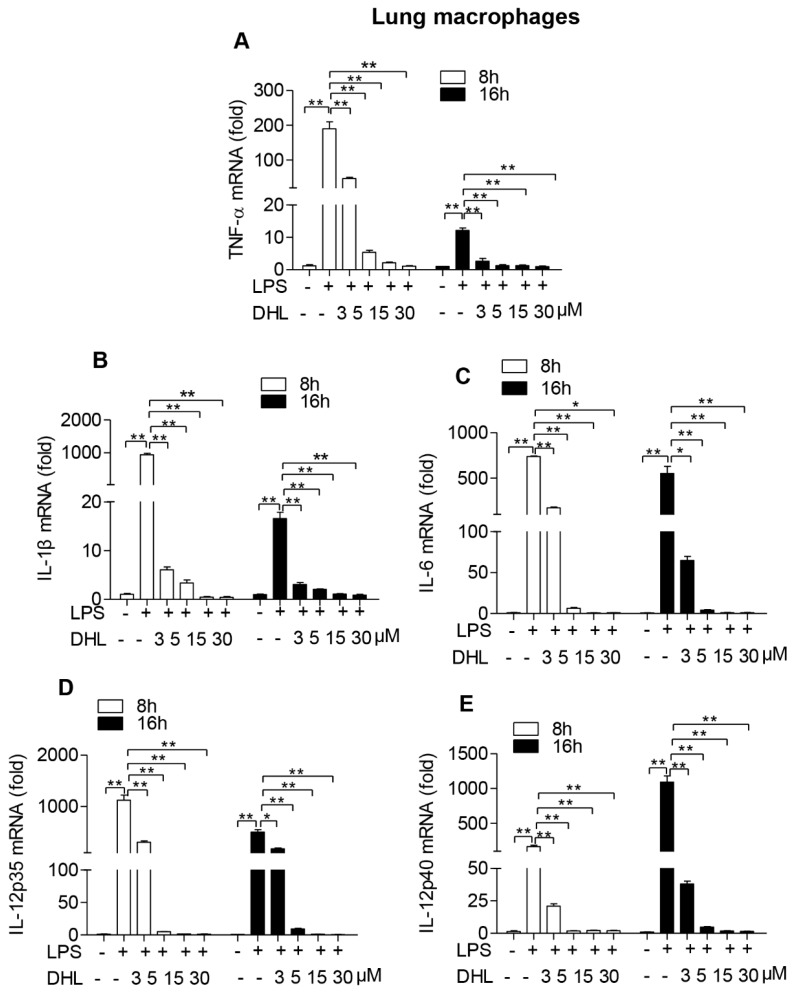
DHL decreased proinflammatory cytokines production in LPS-stimulated lung macrophages. Lung macrophages were pre-treated with 0, 3, 5, 10 and 30 μmol/L DHL for 0.5 h before LPS stimulation (100 ng/mL) for 24 h, cells were collected to isolate total RNAs, the mRNA expression level of (**A**)TNF-α, (**B**) IL-1β, (**C**) IL-6, (**D**) IL-12 p35 and (**E**) IL-12 p40 were measured by quantitative PCR respectively. Values represent means ± SEM, * *p* < 0.05 and ** *p* < 0.01.

As shown in Figure 3A–E, activation with LPS induced significant production of the proinflammatory cytokines TNF-α, IL-1β, IL-6 and IL-12, while pre-incubation with DHL strongly inhibited their mRNA levels in RAW 264.7 cells. The same response to DHL treatment was seen in primary lung macrophages (Figure 4A–E).

### 2.4. DHL Suppressed LPS-Induced Phosphorylation and Nuclear Translocation of NF-κB in RAW 264.7 Cells

NF-κB is the crucial downstream molecule of the P38 MAPK/MK2 and Akt signaling pathways and plays a critical role in regulating cytokine expression in macrophages. To further elucidate the mechanism of DHL’s anti-inflammatory effect we examined activation of NF-κB by detecting phosphorylation and nuclear import of NF-κB p65. 

**Figure 5:**
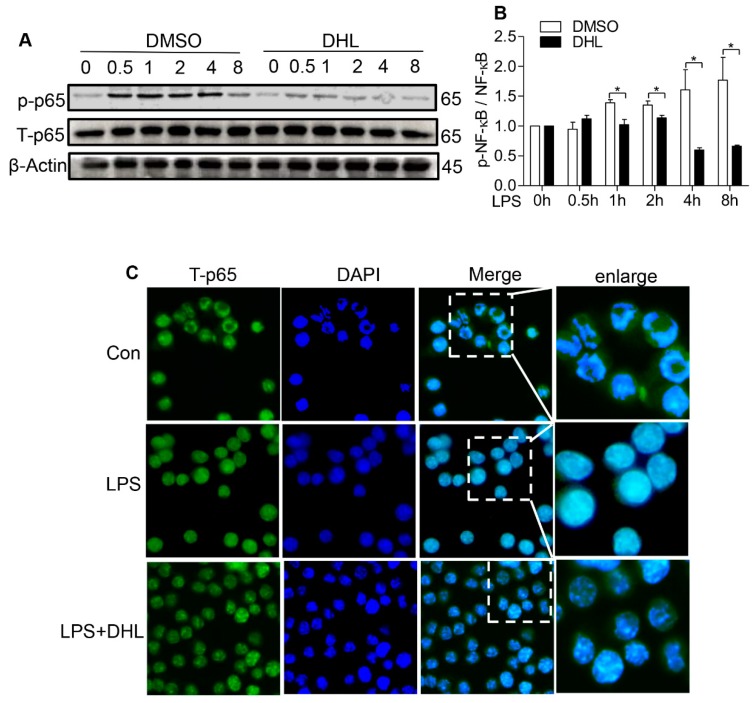
DHL reduced the LPS-induced NF-kB activation in RAW264.7 cells. (**A**) RAW264.7 cells were pre-treated with or without DHL (5 μM) for 0.5 h before LPS stimulation (100 ng/mL) for 0, 0.5, 1, 2, 4 and 8 h. Cell lysates were analyzed by western blot. (**B**) Analysis of the ratios of phosphor-p65 (p-p65) and total-p65 (t-p65) was performed using Image J software. (**C**) RAW264.7 cells were pre-incubated with 5 μM DHL for 0.5 h before challenged with LPS (100 ng/mL) for 0.5 h. Translocation of the p65 subunit of NF-kB (Green) and nuclei (blue) were detected by immunofluorescence assay as described in Materials and Methods. Data represents means ± SEM based on three independent experiments, * *p* < 0.05 and ** *p* < 0.01.

The western blot data showed that DHL decreased p65 phosphorylation induced by LPS (Figure 5A,B). Next, we examined the effect of DHL on LPS-induced NF-κB activation by immunofluorescence assay. As shown in Figure 5C, DHL pretreatment reduced LPS-induced nuclear translocation of NF-κB p65 subunit at 0.5 h. 

### 2.5. DHL Attenuated LPS-Induced Acute Lung Injury

**Figure 6:**
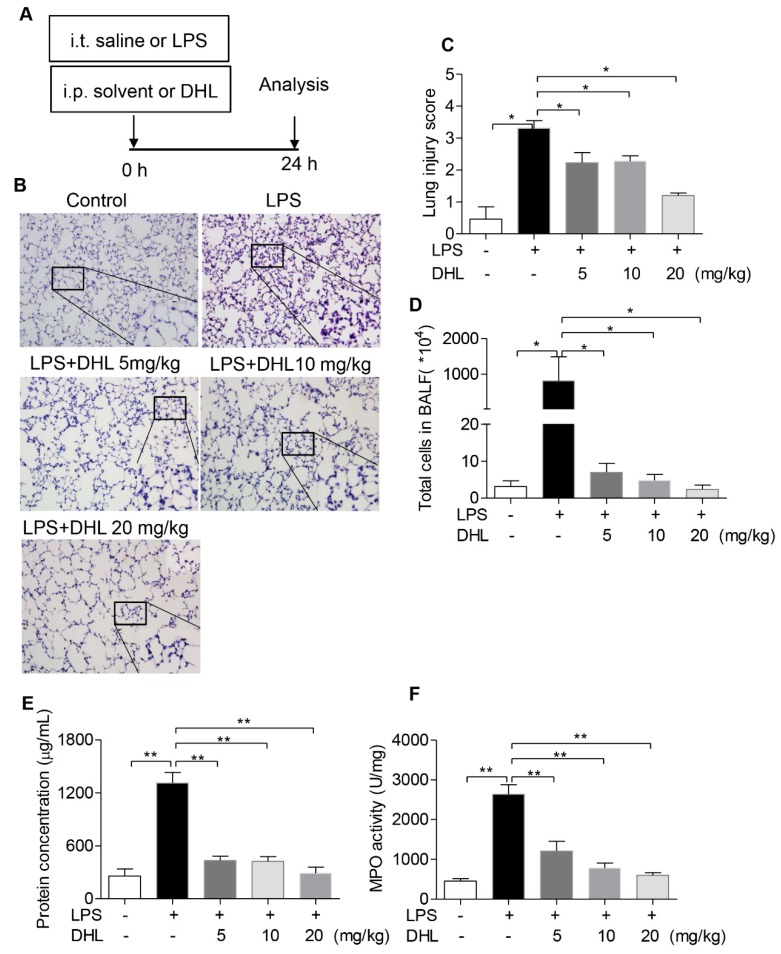
DHL attenuated LPS-induced acute lung injury in vivo. (**A**) Mice were subjected to an intratracheal injection of LPS (5 mg/kg) and subsequent intraperitoneal injection of solvent or DHL (5, 10 and 20 mg/kg respectively), (**B**) Histological analysis of lung tissue sections by H&E staining. The original magnification was 200×, and detailed images are shown in lower right corner. (**C**) Lung tissue injury was assessed by histological scores in all groups. (**D**) The total cell count in BALF after red blood cell lysis. (**E**) Measurement of the total protein concentration of BALF. (**F**) Determination of the myeloperoxidase (MPO) activity in lung homogenates. Data represent means ± SEM, *n* = 6–8, * *p* < 0.05 and ** *p* < 0.01.

To evaluate the therapeutic role of DHL in endotoxin-induced pneumonia in a mouse model, ALI was induced with LPS exposure, followed by intraperitoneal administration of solvent or different concentrations of DHL. The experimental procedure is shown in Figure 6A. The pathological changes in lung tissues were analyzed by H&E staining. The results showed that treatment with DHL effectively reduced the LPS-stimulated infiltration of inflammatory cells and thickening of the alveolar walls (Figure 6B). The degree of lung injury was further determined by a semi-quantitative scoring method [26]. As shown in Figure 6C, DHL alleviated LPS-induced lung injury scores significantly. Lung alveolar permeability was evaluated based on protein concentration and cell counts in bronchoalveolar lavage fluid (BALF). As expected, treatment with DHL (5, 10 and 20 mg/kg) obviously decreased the cell counts (Figure 6D) and total protein concentration (Figure 6E) in BALF. 

Myeloperoxidase (MPO) activity in lung tissue is a specific marker of neutrophil infiltration and indicates inflammatory injury of the lung. DHL treatment reduced the increased MPO activity induced by LPS challenge (Figure 6F). Taken together, these results suggest that DHL protected against LPS-induced lung injury in mice.

### 2.6. DHL Reduced the Production of Proinflammatory Cytokines in LPS-induced ALI via p38 MAPK/ Akt/ NF-κB Phosphorylation

**Figure 7:**
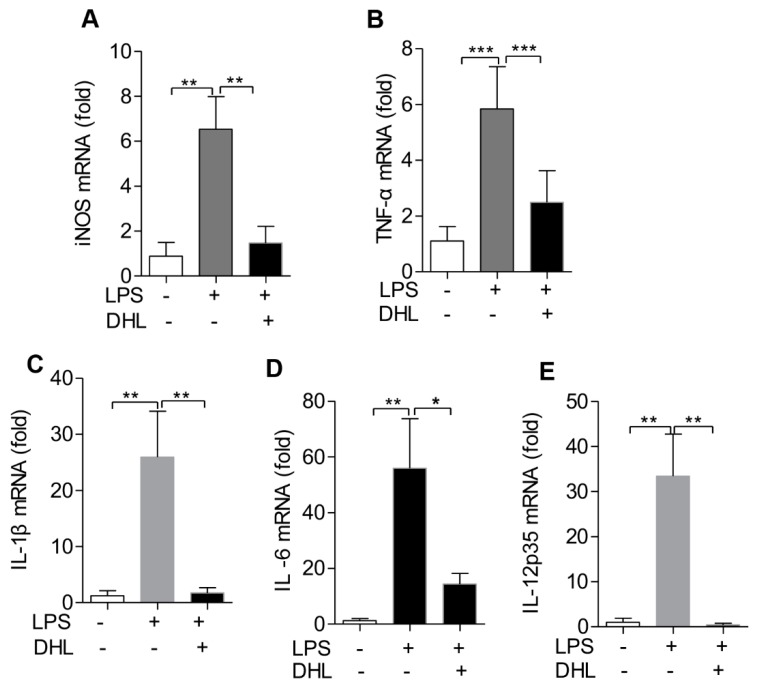
DHL decreased proinflammatory cytokines production in mice treated with LPS for 24 h. Mice were subjected to an intratracheal injection of LPS (5 mg/kg) and subsequent intraperitoneal injection of solvent or DHL (5 mg/kg), then mice were euthanized and the lung tissues were collected to isolate total RNAs. The mRNA expression level of (**A**) iNOS, (**B**) TNF-α, (**C**) IL-1β, (**D**) IL-6 and (**E**) IL-12 p35 were measured by real-time PCR. Data represents means ± SEM, *n* = 6–8, * *p* < 0.05, ** *p* < 0.01 and *** *p* < 0.001.

To further investigate the anti-inflammatory activity of DHL, we used quantitative PCR to detect the expression of proinflammatory cytokines in the lungs of ALI mice with or without DHL treatment. As shown in Figure 7A–E, when compared to the LPS group, the mRNA expression levels of iNOS, TNF-α, IL-6, IL-12 and IL-1β in lung tissues were significantly reduced in the DHL (5 mg/kg) treated group. 

**Figure 8:**
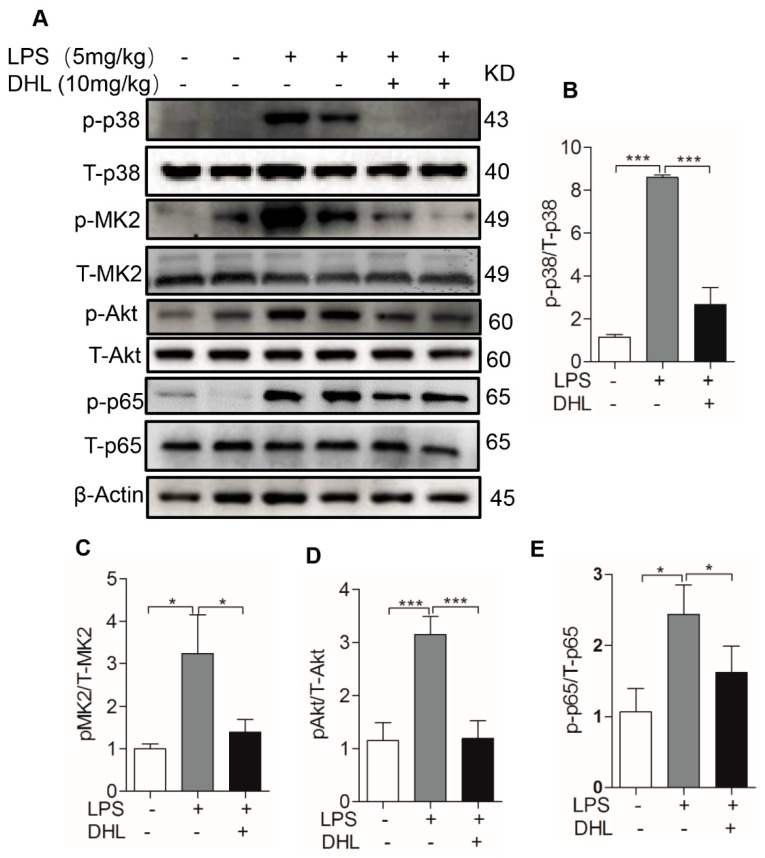
Phosphorylation of p38 MAPK/MK2, Akt and NF-kB involved in the anti-inflammation of DHL in vivo. Mice were subjected to an intratracheal injection of LPS (5 mg/kg) and subsequent intraperitoneal injection of solvent or DHL (5 mg/kg), then euthanized and the lung tissues were collected to measure protein expression. (**A**) The expression levels of specific proteins were evaluated by western blotting. (**B**–**E**) The quantitative analysis of the ratios of p -p38/T-p38, p-MK2/MK2, p-Akt/Akt, and p-p65/p65 normalized with β-actin, was performed using ImageJ software. Data represents means ± SEM, *n* = 6–8, * *p* < 0.05, ** *p* < 0.01 and *** *p* < 0.001.

We next investigated whether LPS-induced expression of inflammatory cytokines in lungs was mediated via NF-κB signal molecule mediated by phosphorylation of p38 MAPK/ MK2 and Akt. The data showed that treatment with DHL significantly suppressed the phosphorylation of Akt, p38 MAPK, MK2 and NF-κB in the lungs of mice with LPS-induced ALI (Figure 8A–E). The in vivo results suggest that DHL alleviated LPS-induced inflammatory responses, and that modulation of p38 MAPK/MK2, Akt and NF-κB were involved in these anti-inflammatory effects.

## 3. Discussion

Inflammation is generally recognized as a defensive response against various aggressions including microbial infections, however excessive inflammation can often cause extensive tissue injury, acute respiratory failure, systemic dysfunction and even death [27]. ALI/ARDS is a major source of morbidity and mortality in patients in intensive care units [28]. Enhanced inflammatory responses with inflammatory cells infiltration and elevated proinflammatory cytokines expression in the lung are key factors in the pathogenesis of ALI/ARDS [29]. There is an increasing body of evidence suggesting that macrophages play a crucial role during the inflammatory response. They act as the first line of defense against infections, releasing various proinflammatory mediators such as TNF-α, IL-1β and IL-6, and also help recruit the neutrophils from the intravascular space into the lungs, which finally leads to the tissue damage seen in ALI/ARDS [5]. Drugs against macrophage-mediated inflammatory response could therefore be an effective option for ALI treatment. 

With the development of pharmacology research, numerous therapeutic components derived from herbal extracts have been identified [30,31]. Sesquiterpene lactones are a large family of compounds which have been shown to exhibit antimicrobial, antitumor, and anti-inflammatory effects [32,33]. Through the years, many of SLs have been reported to be effective on treatment of inflammation. For example, isoalantolactone, a sesquiterpene lactone extracted from roots of *Inula helenium* L, was identified to suppress LPS-induced lung injury and proinflammatory cytokine expression [10]; budlein A can reduce the proinflammatory cytokines and neutrophil recruitment in a model of acute gout arthritis [34]. DHL is a sesquiterpene lactone which has been extensively reported to exhibit anti-tumor and hepatoprotective activities [20,21,35], while the effect of DHL on ALI is rarely examined. Here, we found that DHL reduced the activation of p38 MAPK/MK2 and Akt in macrophages, all of which are the critical downstream signaling molecules of TLR4/MyD88 to stimulate expression of cytokines in LPS-induced inflammation [28]. Compounds that can down-regulate activation of p38 MAPK and Akt signaling molecules are therefore expected to be beneficial in protecting against inflammatory diseases. The inhibition of related signaling molecules by DHL plus anti-inflammatory feature of the whole SLs’ pharmacological profile prompt us to suppose that DHL may be another compound of SLs to exert anti-inflammatory response by regulating macrophage function. 

NO is an inflammatory marker that is used to predict the progression of inflammation and is produced by iNOS in macrophages in response to LPS exposure [36]. We first measured the NO production in macrophages with or without DHL treatment, and found that DHL inhibited the production of NO in both primary lung macrophages and murine macrophage cell line (RAW264.7 cells). Accordingly, our data showed that the mRNA levels of iNOS were significantly increased by LPS stimulation and significantly decreased by DHL treatment. 

Activated macrophages were also shown to secrete high amounts of proinflammatory cytokines such as TNF-α, IL-1β, IL-6 and IL-12. These proinflammatory cytokines can influence the complex signaling network involved in mediating the inflammation process [37]. For example, TNF-α and IL-1β promote the accumulation of neutrophils and the release of other cytokines, initiating and perpetuating the inflammatory response [38,39]. IL-6 and IL-12 maintain tissue homeostasis and control the extent of the inflammatory response [40]. In this study, we found that DHL treatment significantly suppressed the LPS-induced expression of proinflammatory cytokines TNF-α, IL-1β, IL-6, IL-12p35 and IL-12p40. These effects on cytokine expression and macrophage activation indicate that DHL can have an anti-inflammatory influence on endotoxin-induced inflammation. The mouse model of LPS-induced lung injury has been used extensively for exploring potential therapeutic strategies against ALI [41]. Local administration of LPS into the lung by intratracheal injection increases the alveolar epithelial permeability and inflammatory cell infiltration, this in turn results in an accumulation of inflammatory cytokines in BALF and lung [42], which closely resembles the pathology ALI/ARDS in humans [43,44]. Thus, we employed this mouse model to investigate the potential protective effect of DHL on LPS-induced ALI. Our results have shown that DHL treatment significantly reduced LPS-induced pulmonary pathological changes, inflammatory cells infiltration and release of proinflammatory cytokines in lung tissues. These results provide convincing evidence of DHL for treating endotoxin-induced ALI.

One of the most studied mechanisms for the SLs-regulated inflammatory disease is NF-κB, a transcription factor which plays a pivotal role in the transcriptional regulation of various inflammation-related genes [45]. Inactivation of NF-κB was found to result directly in a decreased pro-inflammatory mediator production including TNF-α, IL-6, IL-12 and IL-1β, all indicators of a severe inflammatory response [46]. Importantly, NF-κB is the key target of p38 MAPK and Akt signaling pathways after LPS binding TLR4/MYD88 to initiate intracellular pathways [11,12]. Both p38 MAPK/MK2 and PI3K-Akt can regulate LPS-induced gene expression by controlling NF-kB p65 hyperphosphorylation and nuclear translocation [47,48,49]. Here, our data showed that DHL pre-incubation suppressed NF-κB (p65) phosphorylation and nuclear translocation in LPS-stimulated macrophages. These results suggested that the anti-inflammatory effects of DHL was associated with the inactivation of NF-κB, and the mechanism of DHL in the protective role in LPS-induced ALI may be that DHL treatment reduced activity of NF-κB mediated by both p38 MAPK/MK2 and Akt signaling pathways, resulted in decreased proinflammatory cytokines in macrophages, and hence suppressed LPS-stimulated lung injury. Taken together, DHL suppresses LPS-induced acute lung injury and macrophage activation via inactivating NF-κB, which can be mediated by p38 MAPK and Akt signaling pathway. However, it has been demonstrated in some previous studies that Akt signaling pathway can also be directly or indirectly activated by p38 MAPK/MK2 signaling pathway, and plays a crucial role in the inflammatory response [50]. Thus there may be cross-talk between p38 MAPK/MK2 and Akt signaling pathways, this exact cross-talk effect should be studied further.

## 4. Materials and Methods

### 4.1. Material and Reagents

Dehydrocostus Lactone (C_15_H_18_O_2_, MW,230.3, purity > 98%) was purchased from Target Molecule Corp. (Target Mol, Shanghai, China). LPS (*Escherichia coli*, serotype 055:B5), collagenase and DNase were purchased from Sigma Chemical Co. (St Louis, MO, USA). Dulbecco’s modified Eagle’s medium (DMEM) and fetal bovine serum (FBS) was purchased from Gibco (Thermo Fisher Scientific, Waltham, MA, USA). The bicinchoninic acid (BCA) protein assay kit, NO assay kit (Griess reagent), and phenylmethylsulfonyl fluoride (PMSF) were purchased from Beyotime Biotechnology (Shanghai, China). Primary antibodies against p-Akt(S473), Akt, p-p65 (S536), p65, p-p38, p38, p-MK2, MK2 and β-actin were purchased from Cell Signaling Technology (Danvers, MA, USA). 

### 4.2. Animals

C57BL/6 mice (male, 6–8 weeks old) were ordered from Nanjing Model Animal Research Center (license No. SCXK (Su) 2017-0052) and housed in specific pathogen-free conditions with 12 h light–dark cycles at the Laboratory Animal center of Jiangnan University. All procedures involving the use of the mice were carried out in accordance with the Statute on the Administration of Laboratory Animals by the National Science & Technology Council of China. The experimental study protocals described in this study were approved by the Institutional Animal Care and Use Committee of Jiangnan University, and the ethic aggreement number is JN.No20171115c1001120-68.

### 4.3. Cell Culture and Treatment

The lung macrophages were obtained from male C57BL/6 mice as previously described [51]. Briefly, mice were killed and the lung lobes were excised. Macrophages were isolated from the whole lungs of mice via collagenase digestion (collagenase [1.0 mg/mL], DNase [25–50 U/mL]). Cells were collected and cultured in DMEM plus 10% FBS at 37 °C in a humidified atmosphere incubator with 5% CO_2_ for 1 h, then the nonadherent cells were washed away. The adherent cells were treated with the indicated concentrations of DHL and/or LPS according to the experimental requirements. The murine macrophage cell line RAW264.7 (ATCC, Manassas, VA, USA) cells were cultured in DMEM high glucose medium containing antibiotics (100 units/mL of penicillin and 100 μg/mL streptomycin) and 10% FBS at 37 °C in a humidified atmosphere incubator with 5% CO_2_. Cells were treated with the relative concentration of DMSO or DHL 30 mins before LPS stimulation (100 ng/ mL) according to the implying time.

### 4.4. Cell Viability Assay

Cell viability was determined by MTT assay as previously described [25]. RAW264.7 cells were cultured in 96-well plates at a density of 10^5^ cells/mL and exposed to different concentrations of DHL (3, 5, 15, 30 μmol/L) or 0.1% DMSO as control for 48 h and 10 μL of MTT (5 mg/mL) was added to each well and incubated for 4 h. The supernatant was discarded and the formation was resolved with 150 μL/well of DMSO. The absorbance at 570 was measured on a microplate reader (BioTek Epoch, Winooski, VT, USA). Concentrations were determined for three wells of each sample, and each experiment was performed in triplicate.

### 4.5. Determination of NO Production

RAW264.7 cells and lung macrophages were seeded in 12-well plates at 5 × 10^5^ per well and incubated overnight. Cells were treated with DHL at different concentrations for 30 mins before being challenged with LPS (100 ng/mL) for indicated time. Supernatants were collected and the release levels of NO were measured using the Nitric Oxide Assay Kit (Beyotime, Shanghai, China) by a microplate reader (Flex Station 3; Molecular Devices, Silicon Valley, CA, USA) at 540 nm.

### 4.6. RNA Isolation, Reverse Transcription and Quantitative PCR

RAW264.7 cells (5 × 10^5^) were challenged with LPS for 24 h followed incubation with DHL (0, 3, 5, 15 and 30 μmol/L) for 30 mins. The lung macrophages were challenged with LPS for 8 h and 16 h followed incubation with DHL (0, 3, 5, 15 and 30 μmol/L) for 30 mins. Lung tissues from LPS induced mice model with or without DHL treatment were collected and homogenized. Total RNA were extracted using TRIzol reagent (Corning, Shanghai, China). cDNA was was synthesized by ReverTra Ace qPCR RT Kit (Toyobo, Osaka, Japan) and amplified by realtime PCR on a StepOne Plus system (Thermo Fisher Scientifc, Waltham, MA, USA) with primer sets for iNOS (forward, 5′-CCCTTCAATGGTTGGTACATGG-3′; reverse, 5′-ACATTGATCTCCGTGACAGCC-3′), TNF-α (forward, 5′-TTCTCATTCCTGCTTGTGG-3′; reverse, 5′-ACTTGGTGGTTTGCTACG-3′), IL-6 (forward, 5′-CCACCAAGAACGATAGTCAA-3′; reverse, 5′-TTTCCACGATTTCCCAGA-3′), IL-12p35 (forward, 5′- GGACCAAACCAGCACAT-3′; reverse, 5′- CGCAGAGTCTCGCCATTA-3′), IL-12p40 (forward, 5′- TGAACTGGCGTTGGAAG-3′; reverse, 5′- GAAGTAGGAATGGGGAGTG-3′), IL-1β (forward, 5′-CCAGCTTCAAATCTCACAGCAG-3′; reverse, 5′-CTTCTTTGGGTATTGCTTGG GATC-3′), and GAPDH (forward, 5′-TGCGACTTCAACAGCAACTC-3′; reverse, 5′-CTTGCTCAG TGTCCTTGCTG-3′). The relative expression (defined as fold change) of target gene was given by 2-△△Ct and normalized to the GAPDH. 

### 4.7. Western Blot Analysis

Cells were plated in 6-well plates at 1 × 10^6^/well overnight and then cells were treated with DHL (5 μM), 0.1% DMSO as control for 0.5 h before challenged with 100 ng/mL of LPS (100 ng/mL) as indicated. Cells were collected and lysed in loading buffer (175 mM Tris-HCl, 4.0% SDS, 100 mM DTT, 7.5% glycerin and 0.2% bromophenol blue in ddH_2_O). Lung tissues were collected and lysed in RIPA buffer (Beyotime Biotechnology) plus 1 mmol/L PMSF. Proteins were subjected to SDS/PAGE gels, transferred to polyvinylidene difluoride (PVDF) and incubated with indicated primary antibodies overnight at 4 °C followed by incubation with secondary antibodies for 2 h at room temperature. Quantification of western blots was performed with Image J software (National Institutes of Health, Bethesda, MD, USA).

### 4.8. Immunofluorescence Assay

Raw 264.7 cells (5 × 10^4^/well) were plated in 24-well plates containing cover slips overnight and then were treated with DHL (5 μM), 0.1% DMSO as control for 30 mins before being induced with 100 ng/mL LPS for 0.5 h. The cells on cover slips were then fixed in 4% paraformaldehyde for 10 min and permeabilized with 0.1% Triton X-100. After blocking in 5% BSA for 1 h, cells were incubated with primary antibodies against NF-κB p65 overnight at 4 °C. After removing the primary antibodies and triplicate washes with PBS, the samples were further incubated with Alexa 488-labeled secondary antibody (CST, Danvers, MA, USA) for 2 h at room temperature. Finally, the samples were incubated with DAPI (2.5 μg/mL) for nuclear staining and photographed under a laser-scanning confocal fluorescence microscope (TCS SP8, Leica Microsystem, Wetzlar, Germany).

### 4.9. ALI Model

LPS was dissolved in PBS, and DHL was dissolved in a solvent (H_2_O:ethanol:polyoxyethylene hydrogenated castor oil = 8:1:1). After acclimation for around 2 weeks under a specific pathogen-free environment (25 °C, 55% humidity, with adequate food and water), the 8- to 10-week-old male C57/BL6 mice (20 ± 3 g) were randomly divided into 5 groups: LPS-induced ALI models were generated by intratracheal administration with of LPS (5 mg/kg), while some groups were further received DHL intraperitoneally as a therapeutic agent in in a range from 5 to 20 mg/kg. body Mice were euthanized 24 h after LPS administration, lung tissues and BALF were collected for analysis.

### 4.10. Histopathological Analysis

Lung tissues were fixed by 4% paraformaldehyde, embedded in paraffin and cut into 4-μm-thick sections. Slices were stained with hematoxylin and eosin(H&E), and images were randomly captured by a microscope (RM2235; Leica Biosystems). The degree of lung injury was assessed and graded from 0 (normal) to 4 (severe) in four categories as previously described in a blinded manner [24]: no injury, 0: injury to 25% of the field, (1) injury to 50% of the field, (2) injury to 75% of the field, (3) and diffuse injury, (4) The total lung injury score was calculated by adding up the individual scores of each category and 6-8 mice from each group were used for histomorphometric scoring.

### 4.11. BALF Analysis

The BALF was collected by lavaging the lung with 1 mL PBS two times and the red blood cells were lysed using lysis buffer followed by centrifugation. The remaining cells were washed, resuspended with PBS and counted, and the supernatant was harvested for protein concentration assay using the BCA protein assay kit (Beyotime Biotechnology).

### 4.12. MPO Activity Assay

The lung tissues were collected and homogenized in 0.5% hexadecyl trimethyl ammonium bromide (HTAB) diluted in 50 mM phosphate buffer. After centrifugation, the pellets were resuspended in 0.5% HTAB and subjected to a freeze-thaw process three times. Collected the supernatants and determined the protein concentration by the BCA protein quantitation kit. Next, 3,3′,5,5′-tetramethylbenzidine and H_2_O_2_ were added to the supernatant, followed by measuring of the absorbance at 655 nm for 5 min. The MPO activity was calculated as the absorbance change per min per gram protein [52].

### 4.13. Statistical Analysis

The vitro data were expressed as means ± SEM obtained from at least three independent experiments. The in vivo data were expressed as means ± SEM obtained from experiments by at least 6-8 mice in each group. All statistical analyses for single comparisons were performed by Student’s t-test, and the differences between the groups were performed by the One Way ANOVA multiple comparisons test using GraphPad Prism 5 (GraphPad Inc., San Diego, CA, USA). For all analyses, the results were considered statistically significant at *p* < 0.05.

## 5. Conclusions

DHL, a sesquiterpene lactone, could inhibit the production of inflammatory mediators in LPS-stimulated macrophages. The inhibitory effect of DHL was associated with the downregulation of p38MAPK and Akt phosphorylation, suppressing the activation of NF-κB. DHL could also attenuate inflammatory responses in the LPS-induced ALI mice model. Our results suggest that DHL may serve as a promising agent in the treatment of endotoxin-induced ALI.
